# Supplementary figures and images for: High Variation in Protist Diversity and Community Composition in Surface Sediment of Hot Springs in Himalayan Geothermal Belt, China
Source: Microorganisms. 2023 Mar 7;11(3):674. doi: 10.3390/microorganisms11030674 (PMC10053680; doi:10.3390/microorganisms11030674)

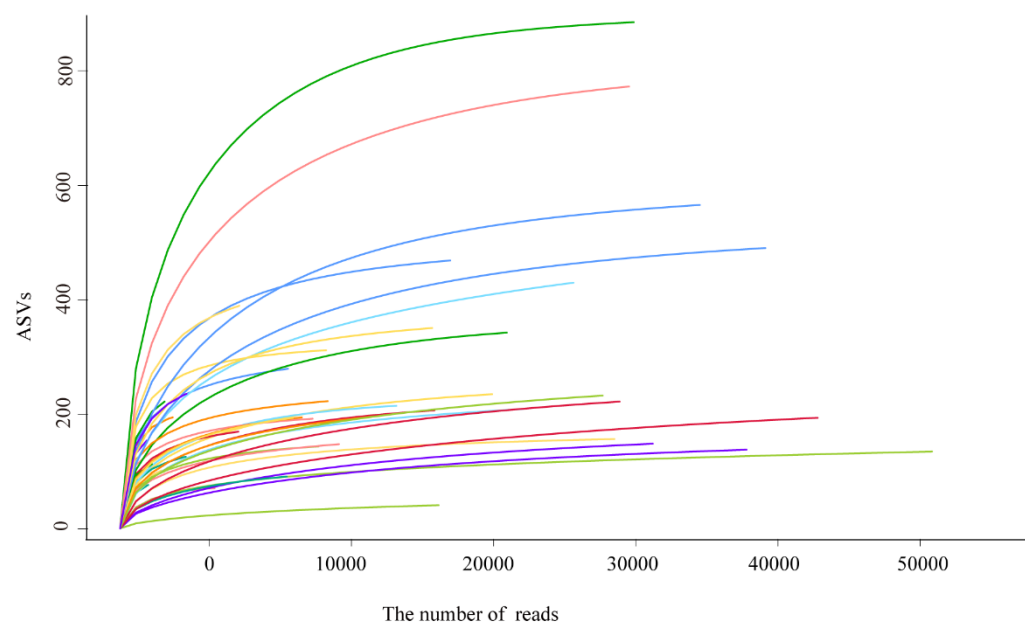

Figure S1. Rarefaction curves for protists in hot springs in HGB

Supplement: Supplementary file 1 [file microorganisms-11-00674-s001.zip › Supplementary S1.pdf]
